# Effect of Media Warnings on Rabies Postexposure Prophylaxis, France

**DOI:** 10.3201/eid1706.101962

**Published:** 2011-06

**Authors:** Philippe Gautret, Caroline Labreuil, Mohamadou Seyni, Jean Delmont, Philippe Parola, Philippe Brouqui

**Affiliations:** Author affiliation: Hôpital Nord, Assistance Publique-Hôpitaux de Marseille, Marseille, France.

**Keywords:** rabies, viruses, zoonoses, media, prophylaxis, France, letter

**To the Editor:** Rabies was officially declared eliminated in nonflying mammals in metropolitan France during 2001–2008 by the World Organisation for Animal Health; the last case of rabies was believed to occur in a fox in 1998. However, rabies remained a public health concern because of the risk of translocation of infected dogs from enzootic areas and the natural circulation of bat rabies–associated lyssaviruses (BRALVs). In 2008, France temporarily lost its rabies-free status following evidence of an indigenous case of rabies in a dog, linked to an index case in a dog infected in Morocco. In 2007, a domestic cat was found infected with a BRALV, an indication that, although bats are the primary hosts of this pathogen, other mammals may be infected (*1*). Rabies is a tragic and frightening disease, and bats have a sinister image. Therefore, possible transmission of rabies from bats to humans represents a particularly terrifying threat in which emotional distortion may play a key role in public responses. Patient demand for rabies postexposure prophylaxis (RPEP) has been associated with media-communicated health alerts in France (*2**,**3*) and French Guiana (*4*).

We compared the number of RPEP treatments in humans after bat-related exposures in the south of France with newspaper reports about rabies-related events over an 8-year period. In France, primary health care management of patients seeking RPEP is delivered through an official network of antirabies medical centers. All centers in the southern half of France were asked to provide the number of RPEP treatments that followed bat-related exposures in mainland France during 2002 through 2009. Of 22 centers, 18 participated in the study, reporting 326 RPEP treatments (Figure). Two marked peaks were observed: in September 2004 and in September 2008. The number of patients reporting bat-related exposures that occurred during the summer period (June–September) showed marked annual variations with a 2.1-fold increase in 2004 (44 cases) and a 4.7-fold increase in 2008 (96 cases) compared with the 2002–2009 average of 20.5 cases/summer (range 7–31). Most cases in 2008 were reported by the Marseille and Bordeaux centers. In 2004, 3 cases of illegally imported dogs with rabies were observed in France, in February, May, and August (*2**,**5*). Newspapers reported extensively on the third case, with 54 articles published in the 3 major national newspapers (Le Monde, Le Figaro, Libération) (*2*), after an alert was issued in late August (Figure). On July 30, 2008, in response to a familial cluster of RPEP following bat bites near Marseille, a media-communicated health alert was organized by the Marseille center in the major regional newspaper (La Provence) to warn people about the potential risk of rabies after bat-related exposures and the necessity of seeking advice when such events occur. The alert was developed by the Agence France Presse, released by the majority of national and regional newspapers, and included in a large number of websites (Figure). On August 28, 2008, an additional alert was released in Bordeaux because of 2 BRALV-infected bats in the region (Figure). The alert was published in the regional newspaper (Sud-Ouest) (*3*). No other rabies-related events were intensively reported in French media during the study period.

**Figure:**
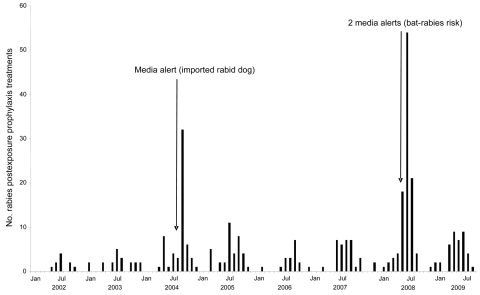
Number of rabies postexposure prophylaxis treatments caused by bat-related exposures as reported by 18 antirabies medical centers in southern France, by time of first visit, 2002–2009. Centers responding were Annecy, Annonay, Aurillac, Bastia, Bordeaux, Chambéry, Grenoble, Le Puy en Velay, Limoges, Lyon, Marseille, Nice, Pau, Perpignan, Poitiers, Roanne, Saint-Etienne, and Toulouse.

The pattern of spikes in RPEP in the south of France seen after bat-rabies media reports supports the results of other studies that found effective newspaper reporting increases patient demand for RPEP (*2**–**4*). However, the increase is of short duration. Our results may indicate that media reports bring out the worried well who were not truly exposed but still request RPEP. The results may also indicate that bat-related injuries or contacts are underreported in the absence of media events.

Classical rabies virus has been transmitted to humans by bats in South America (*6*). In Europe, European bat lyssavirus type 1 (EBLV-1) has been isolated from bats in Germany, France, the Netherlands, Denmark, Yugoslavia, Spain, and Poland and EBLV-2 from bats in the Netherlands, United Kingdom, Switzerland, Germany, and Finland (*6**,**7*). Only 3 cases of rabies in humans caused by bat bites have been reported in Europe (*6*). In France, health authorities recommend that all cases of confirmed bat-related exposures (bites, scratches, exposures to a mucous membrane) receive RPEP with both vaccine and immunoglobulin (*8*), based on World Health Organization guidelines (*9*). Whether RPEF is needed for other types of exposures is subject to considerable debate (*10*). The centers in southern France are directed to provide RPEP only for known exposures.

Based on results of study we conducted in 2008 when RPEP increased 4.7 fold after 2 media releases within several weeks, it is possible that only 20% of persons with bat exposure typically seek RPEP in periods without media reports. Although the risk of human rabies acquired through exposure to European bats is rare, information should be provided to the French public to avoid direct contact with bats, including handling when found inside homes during the summertime. When available, bats should be submitted for rabies testing to determine whether RPEP is needed. Following these procedures should minimize both the potential risk for transmission and the number of expensive RPEP treatments.
